# Successful Treatment of Pulmonary and Cerebral Toxoplasmosis Associated with Pneumocystis Pneumonia in an HIV Patient

**DOI:** 10.3390/diseases5040035

**Published:** 2017-12-18

**Authors:** Marie-Françoise Rey, Charles Mary, Diane Sanguinetti, Stéphane Ranque, Christophe Bartoli, Coralie L’Ollivier

**Affiliations:** 1 Department of Interregional Secure Hospital, AP-HM, University Hospital, 13915 Marseille CEDEX, France; Marie-francoise.REY@ap-hm.fr (M.-F.R.); christophe.bartoli@ap-hm.fr (C.B.); 2 Université Aix Marseille, CNRS 7278, IRD 198, Inserm 1095, AP-HM, URMITE, IHU Méditerranée Infection, 13915 Marseille CEDEX, France; charles.mary@ap-hm.fr (C.M.); diane.sanguinettimorelli@gmail.com (D.S.); stephane.ranque@ap-hm.fr (S.R.)

**Keywords:** *Pneumocystis jirovecii*, *Toxoplasma gondii*, pneumonia, cerebral, trimethoprim/sulfamethoxaxole

## Abstract

In both the post and pre combination antiretroviral therapy (cART) era, *Pneumocystis jirovecii* and *Toxoplasma gondii* remain common opportunistic infectious agents. The common manifestations are pneumonia for *P. jirovecii* and brain abscess for *T. gondii*. Nevertheless, co-infection remains rare, and pulmonary toxoplasmosis is scarce, or may be underestimated because of its similarity with *Pneumocystis jirovecii* pneumonia. We reported an uncommon case of an AIDS patient (6 CD4 + T cells/mm^3^) with both pulmonary and cerebral toxoplasmosis associated with pneumocystis pneumonia. The patient presented with general weakness, fever and dyspnea. Pulmonary toxoplasmosis and pneumocystis were confirmed by microscopic examination and DNA detection in the bronchoalveolar lavage. Computed tomography imaging of the brain revealed a single characteristic cerebral toxoplasmosis lesion of the left capsular area. He was successful treated by trimethoprim/sulfamethoxaxole in conjunction with an early reintroduction of cART, and without IRIS development. During a 3-year follow-up, HIV viral load remained undetectable, and the patient did not relapse for toxoplasmosis or Pneumocystis pneumonia.

## 1. Introduction

In the course of HIV infection, the progressive destruction of CD4 + T cells in the absence of control of viral replication leads, over time, to an increased risk of opportunistic infection. Bacterial pneumonia and *Pneumocystis jirovecii* pneumonia (PCP) remain the leading causes of acute respiratory failure [1,2]. On the other hand, pulmonary toxoplasmosis (PT) is relatively rare, with a 0.5–0.6% prevalence, as estimated in French surveys [2,3]; whereas, cerebral toxoplasmosis (CTX) is the most common opportunistic infections of the central nervous system (CNS), in both the post and pre combination antiretroviral therapy (cART) era [4]. Overall, the risk of toxoplasmosis manifestations resulting from the reactivation of latent tissue cysts is associated with a low CD4 + T cell count (less than 100 cells/µL). The first-line agent for the treatment of PCP is trimethoprim/sulfamethoxaxole (TMP-SMX) (or co-trimoxazole) [1]. The reference treatment for cerebral and pulmonary toxoplasmosis is a combination of pyrimethamine and sulfadiazine, which is systematically combined with folinic acid to prevent the myelotoxicity of pyrimethamine [5]. Alternative therapies are (i) TMP-SMX, (ii) pyrimethamine combinations with clindamycin, atovaquone or azithromycin, or (iii) atovaquone plus sulfadiazine [6]. Here, we report a case of an AIDS patient with both pulmonary and cerebral toxoplasmosis associated with pneumocystis pneumonia, treated by TMP-SMX. 

## 2. Case Description

**Figure 1:**
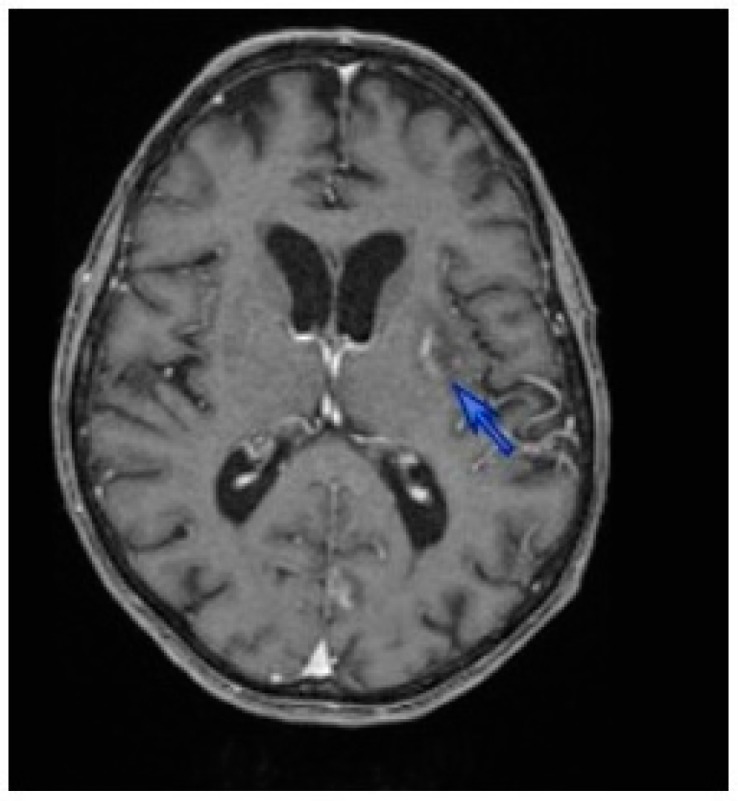
Computer tomography (CT) brain showing a 23 nm lenticular lesion in the left basal ganglia (blue arrow).

In October 2013, a 51-year-old man from South France was hospitalized for 40 kg weight loss caused by a dysphagia due to an oral candidiasis and herpes over the previous four months. He presented with general weakness, fever, productive cough and dyspnea with normal oxygen saturation. He had been diagnosed HIV positive in 2003. For three years, he showed a poor compliance with anti-HIV treatment. There was no history of travel abroad. Laboratory test results showed: 0.51 Giga/L lymphocytopenia with 6 CD4 + T cells/mm^3^; 70.7 mg/L C-reactive protein (CRP) level (N < 10); 681 U/L lactate dehydrogenase (LDH) level (N 100–620); 6.85 mg/L beta 2 micro globulin (N 1.09–2.53) level; and HIV viral load higher than 500,000 copies/mL. *Toxoplasma* serology tested positive, showing an acquired immunity profile (positive IgG and negative IgM antibodies) since 2010. Hepatitis C serology showed a past infection profile. Both computed tomography (CT) and positron emission tomography (PET) scans showed micro-cysts and bilateral diffuse ground-glass opacities in the lungs, and a single-characteristic cerebral toxoplasmosis lesion of the left capsular area (Figure 1). The microscopic examination of Diff Quick and Toluidin Blue-stained bronchoalveolar lavage (BAL) specimen showed *Toxoplasma gondii* tachyzoites, and both trophic and asci forms of *Pneumocystis jirovecii*. *T. gondii* and *P. jirovecii* identification was confirmed by PCR assays targeting the rep-529 gene and 18S rRNA, respectively [7,8]. BAL tested negative for mycobacteria, fungi and CMV. The final diagnosis was pulmonary toxoplasmosis and Pneumocystis pneumonia associated with cerebral toxoplasmosis in a patient with HIV-associated immunosuppression. 

The patient was treated with co-trimoxazole (20 mg/kg trimethoprim and 100 mg/kg sulfamethoxazole per days) for 3 weeks, followed by 800 mg/day co-trimoxazole secondary prophylaxis until immune reconstitution. Concomitantly, antiviral therapy with tenofovir, emtricitabine and lopinavir was reintroduced. The patient’s clinical status quickly improved, and both lung and cerebral CT scan lesions decreased. Nevertheless, within one month, the patient presented with two bloodstream infection (*Staphylococcus aureus* and *Klebsiella pneumoniae*) episodes, which were successfully treated by antibacterials. After 2 months of cART, the CD4 + T cell count rose to 80/mm^3^ and the HIV viral load became undetectable. At this time, the patient was discharged from the hospital. During a 3-year follow-up, HIV viral load remained undetectable, and the patient had not relapsed for toxoplasmosis or Pneumocystis pneumonia. 

## 3. Discussion

A decline in the incidence of AIDS-defining opportunistic infections was found following the introduction of cART, which restores the T cell-mediated immune response [9,10]. Although a drastic decrease has been observed both for non-CNS disease and CNS infections, *P. jirovecii* and *T. gondii* remain common opportunistic infectious agents. However, to our knowledge, reports of patients presenting with combined pulmonary and/or cerebral toxoplasmosis and pneumocystis infection are scarce [11,12]. Pulmonary toxoplasmosis (PT) has become a very rare parasitic infection since the advent of highly active antiretroviral therapies. A study in Taiwan showed that respiratory failure was the leading cause of Intensive Care Unit (ICU) admissions among HIV-infected patients. In this study, 8.1% of the patients admitted to the ICU had been diagnosed with *P. jirovecii* pneumonia; 25.9% of the patients were classified as suffering from interstitial pneumonitis with unknown etiology, and presented with radiographic findings and a clinical response to specific anti-pneumocystis therapy compatible with pulmonary toxoplasmosis [13]. The clinical manifestations and radiographic findings of PT are nonspecific, but in the absence of early diagnosis and specific treatment, its lethality rate was 40% [14]. Usually, *T. gondii* pneumonia manifests with fever, dyspnea and non-productive cough. The most common finding on chest radiographs is bilateral diffuse interstitial infiltrates [3,5]. Finally, diagnosis of PT is based on the identification of tachyzoites or DNA of *T. gondii* in samples of BAL, biopsy specimens, or necropsy. Additionally, the clinical and radiological appearance of TP may be indistinguishable from the far more common *P. jirovecii* pneumonia [14]. It is noteworthy that a blood level elevation of lactic dehydrogenase (LDH) is known to be associated with toxoplasmosis and pneumocystis pneumonia [15], but with a rate higher with *T. gondii* than *P. carinii*. In HIV patients, toxoplasmic encephalitis (TE) is one of the most frequent opportunistic infections. It is estimated that the lifetime risk of an untreated HIV-positive patient for *T. gondii* developing toxoplasmic encephalitis is about 25% [16]. The risk of toxoplasmosis infection depends on the prevalence of *T. gondii* infection in the population. In France, a study has evaluated the incidence of TE at 1.0 cases per 100 person-years (95% CI, 0.9–1.1 cases per 100 person-years) since the availability of cART [17]. TE diagnosis is often presumptive, based on the seropositivity for anti-*Toxoplasma* IgG, a compatible clinical presentation, and typical lesions at the brain imaging. Diagnosis confirmation relies on the adequate response to anti-*Toxoplasma* treatment and the absence of alternate diagnosis [18]. According to the Centers for Disease Control (CDC), the diagnostic criteria of presumptive TE are (i) recent onset of a focal neurologic abnormality consistent with intracranial disease or a reduced level of consciousness; (ii) evidence by brain imaging (computed tomography or nuclear magnetic resonance) of a lesion with a mass effect, or of which the radiographic appearance is enhanced by injection of contrast medium; (iii) positive immunologic tests for toxoplasmosis; or (iv) successful response to therapy for toxoplasmosis [19]. The treatment of pulmonary or cerebral toxoplasmosis are identical [14]. The first-line regimen is a combination of pyrimethamine-sulfadiazine plus folinic acid. TMP-SMX given orally or intravenously has been reported in a randomized trial to be effective and better tolerated than pyrimethamine-sulfadiazine [20]. However, no case series have shown the efficacy of TMP-SMX alone for treating *P. jirovecii* and *T. gondii* co-infection. Bruck et al. [12] summarized the recommendations for the management of combined PCP and TE based on 4 case reports of patients treated by (i) TMP-SMX + pyrimethamine + sulfadiazine, (ii) atovaquone + pyrimethamine + sulfadiazine, (iii) TMP-SMX + pyrimethamine + clindamycin, and (iv) pyrimethamine + clindamycin + primaquine. Clearly, the treatment choice should aim for the lowest toxicity. There is no parenteral formulation of pyrimethamine for patients in whom the oral route is contraindicated. The only widely available parenteral sulfonamide is the sulfamethoxazole component of TMP-SMX [6]. Our patient presented a severe diarrhea that precluded oral treatment; thus the parenteral formulation of TMP-SMX, combining anti-*Pneumocystis* and -*Toxoplasma* activity, was chosen. The patient recovered, and did not relapse within 3 years of follow-up. The improvement under an unconventional therapy might also in part result from the subsequent early commencement of antiretroviral drugs therapy in our patient. cART therapy was introduced immediately after the PCP and TP diagnosis; that to say, five days after patient admission. The optimal timing of initiation of cART in patients presenting with an opportunistic infection (OI) has been a matter of dispute; the major issues being increased adverse outcomes resulting from overlapping toxicities of combined treatments and the risk of immune reconstitution inflammatory syndrome (IRIS) [21]. The identified risk factors for IRIS are: male sex, low CD4 + T cell count at ART initiation, high HIV RNA at ART initiation, being antiretroviral-naive at the time of the OI diagnosis, and a short lag between the OI therapy initiation and the ART initiation [22]. However, since 2009, international guidelines have recommended that HAART should be started as early as possible (except in certain circumstances, such as cryptococcal meningitis), regardless of the baseline CD4 count, regardless of heterogeneous clinical practices, local guidelines, and access to cART [6]. In our patient, early instauration of cART permitted reducing HIV-RNA levels and boosting of his immune system; this may have contributed to the success of TMP-SMX. Although he had several risk factors, he did not develop an IRIS.

## 4. Conclusions

This case suggests that the combination of trimethoprim/sulfamethoxaxole (TMP-SMX) and early reintroduction of cART is an effective treatment for both pulmonary and cerebral toxoplasmosis associated with pneumocystis pneumonia in AIDS patients.
